# Sprengel's Deformity: A Paediatric Case Report

**DOI:** 10.7759/cureus.60330

**Published:** 2024-05-15

**Authors:** Aashita Malik, Sham Lohiya, Jayant D Vagha, Keta Vagha, Shikha Kakkat

**Affiliations:** 1 Paediatrics, Jawaharlal Nehru Medical College, Datta Meghe Institute of Higher Education and Research, Wardha, IND

**Keywords:** sprengel's shoulder, hemivertebra, congenital, omovertebral bone, scapular abnormality

## Abstract

Sprengel's deformity is a conspicuous anomaly, affecting one or both scapulas. The congenital elevation of the scapula is frequently accompanied by additional anomalies, such as rib, vertebral, or muscular deformities, among which are rib fusion or vertebral deformity. Defects in the cervical vertebrae are most likely to result in Klippel-Feil syndrome, which is characterised by a short neck, restrictions on head mobility, and low-growing neck hair. Fewer than half of the instances had scoliosis, which is compensatory due to efforts to straighten the spine. The present case report was the case of Sprengel's deformity reported to our department.

## Introduction

Congenital scapular elevation, sometimes called "Sprengel's shoulder," is a shoulder girdle abnormality that is characterised by aberrant descent and changed scapular position and morphology. The deformity is typically accompanied by muscle hypoplasia or atrophy, and the interaction of these elements causes the shoulder to be disfigured and functionally limited [1]. Sprengel was the first to propose that the anomaly had a congenital origin and described its associated pathology [2]. This malformation received its name from Sprengel's 1891 description of it [3]. This uncommon congenital disorder, whose cause is unclear, results from the scapula's caudal migration being stopped during development. Between the scapula and the cervical spine, a fibrous, cartilaginous, or bony structure (still known as the "omovertebral bone") is interposed in 25 to 50% of instances [4,5]. It is not a purely aesthetic defect and often has functional impairments like limitation of movement and pain [6]. The distinguishing feature is the elevation of the scapula. The present finding is a lump at the back of the neck with limited movement in the arm and/or shoulder. The scapula in question is rotated about its sagittal axis. The superior and vertebral borders are respectively closer to the medial line and the axilla [7]. Prior to surgery, the omovertebral bone or its fibrous or cartilaginous analogue must be removed. Therefore, imaging is crucial to establishing the existence of this omovertebral structure [4]. The present case report was the case of Sprengel's deformity reported to our department.

## Case presentation

**Figure 1:**
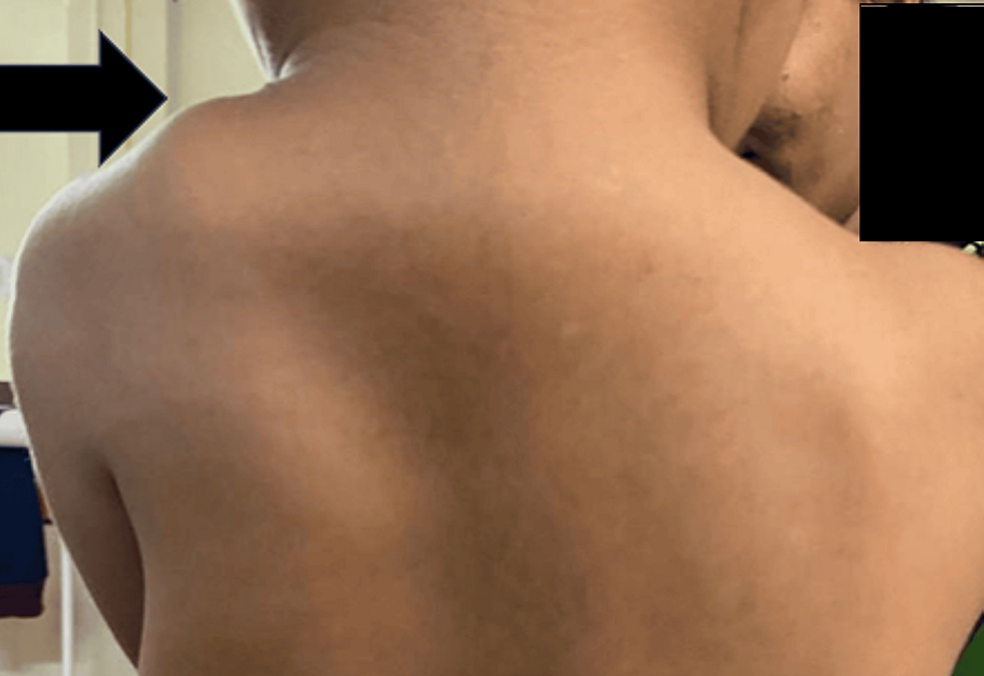
Sprengel's shoulder on the left side

A three-year-old female was brought with a complaint of a high-placed left scapula. As narrated by the mother, she noticed at 10 months of age that her child had left scapula in an abnormal position. There was a noticeable difference between the height of the two shoulders, with the left shoulder being higher as compared to the right shoulder (Figure 1). This disparity increased as the child grew.

There was a complaint of pain on prolonged sleeping in the supine position. There was no restriction of movement of the upper limb, and the child was able to carry out all everyday activities with ease. All developmental milestones were attained according to age. During examination, inspection showed that the left shoulder was higher than the right shoulder. On palpation of the back, the spine of the left scapula was not appreciated, and the left scapula was palpated at a higher level as compared to the right side, hard in consistency. The bulk, tone, power and reflexes of bilateral upper limbs were all normal, and the range of movement was all intact. No abnormalities were detected upon palpation of the spine, and the remainder of the systemic examination revealed no other anomalies. A 2D echo and USG abdomen-pelvis were also done to rule out any other congenital anomalies and both were normal. The Department of Orthopaedics was consulted wherein the child was advised for an X-ray of the neck and chest.

**Figure 2:**
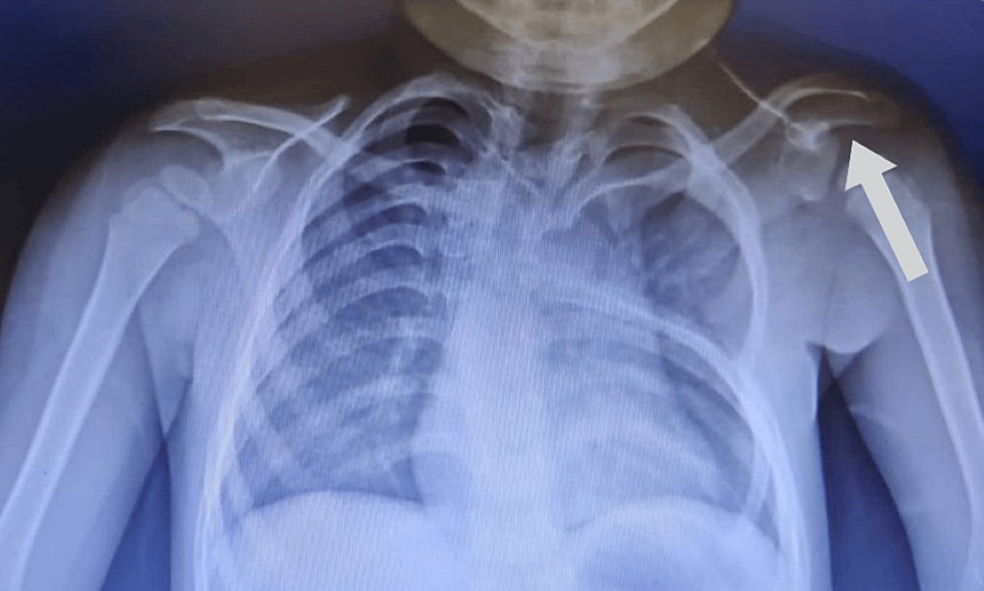
X-ray showing scapula placed at a higher position on the left side

The X-ray findings showed that the scapula was placed at a higher position on the left side, was smaller in size, and was seen to have a bony appendage superiorly (Figure 2).

**Figure 3:**
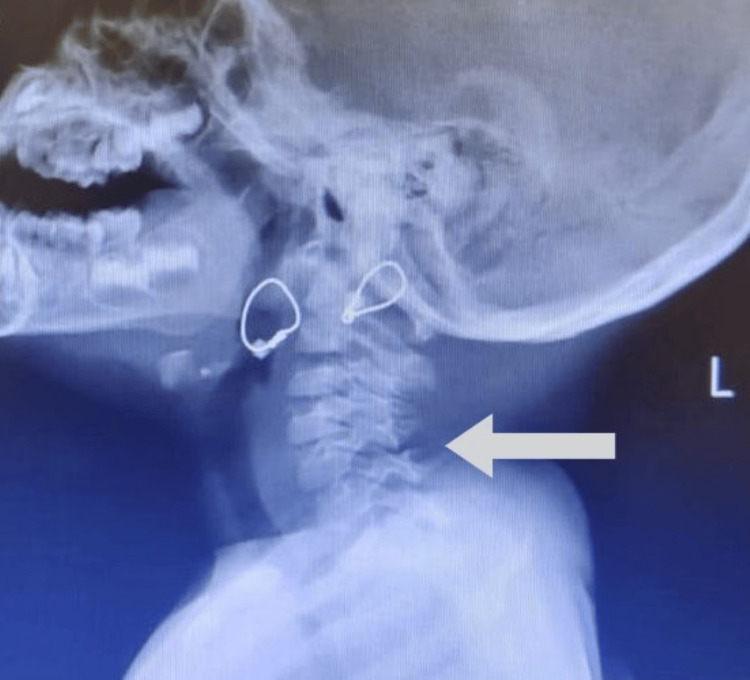
X-ray showing hemivertebra at t2 and t4 levels, with scoliosis in the upper dorsal spine

The X-ray of the upper dorsal spine showed hemivertebra at t2 and t4 levels, with scoliosis in the upper dorsal spine and advised for paediatric surgery (Figure 3).

A review call was sent to the Department of Orthopaedics, wherein they advised only for regular physiotherapy. No orthopaedics intervention was advised from their side. A paediatric surgery was consulted but no intervention was advised from their side. The child was given symptomatic treatment for the management of pain and also taught exercises for adequate and proper movement of the upper limbs.

## Discussion

This condition's physiopathogenesis is still a mystery. In the fifth week of intrauterine life, the scapula typically develops as a mesenchymal mass at the level of the fourth and fifth cervical vertebrae [8]. It migrates caudally starting in the sixth week and reaches its final physiological location in the 12th week. The sixth and eighth thoracic vertebrae are then where the inferior angle of the scapula lies. The scapular shape adapts to the prehensile function of the upper limb throughout this caudal migration [8]. The scapula's morphology begins to resemble that of an adult scapula by the 12th week of intrauterine life, appearing taller than it is wide. The disruption of this caudal movement leads to Sprengel's deformity. Although the exact reason is still unknown, it may be vascular [9,10].

There have been a few familial cases of Sprengel's deformity reported, which raises the prospect of hereditary transmission [10]. It is a fairly uncommon skeletal malformation, according to Sulamaa and Wallgren. The congenital variant is more prevalent, although it can also be acquired. The ratio of bilateral to unilateral cases is 1:10 [11]. Das et al. reported a case of an eight-year-old male child with a bilateral webbed neck more pronounced on the right side [12]. There was a slight degree of abduction of the right shoulder joint. An examination from the back revealed a hypoplastic shoulder more pronounced on the right side. On a plain radiograph, it was seen that the superior border of both the scapula is higher up on the right side. There was lateral bending of the cervical column of vertebrae with convexity on the right side. There was crowding of the transverse processes of the cervical vertebrae, along with fusion of their lateral masses on the left side. There was a decrease in distance between the convex medial borders of both scapulas. The inferior angles of both the scapulas were found at the level of T4 on the right side and T6 on the left side, and the angles were very prominent. On the radiograph, no omovertebral bar was seen. No cardiopulmonary abnormality was seen, and all movements were intact except abduction at the shoulder joint. No other associated anomalies were found [2]. In a case study by Li et al., a teenage girl presented with significantly reduced shoulder abduction caused by untreated severe Sprengel's deformity and subsequently underwent deformity correction surgery [13]. A scapula is elevated and rotated as seen on a simple chest X-ray, and scoliosis needs to be checked out as well. The use of multiplanar and three-dimensional reconstructions in CT is a useful diagnostic tool that directs surgical therapy [14-17]. The presumed diagnosis is supported by the visualisation of a scapula at the predicted location on the child's back in an axial plane, with the contralateral scapula missing since they are not located at the same level. The surgery is only used to cure serious malformations or dysfunctions in children between the ages of three and eight [18]. There are a few cases of Sprengel's deformity with the association of heart disease and renal anomalies [19,20].

## Conclusions

In conclusion, this case report highlights the clinical presentation, diagnosis, and management of Sprengel's deformity, a rare congenital anomaly affecting the shoulder girdle. By using a plain radiograph and a clinical examination, a doctor can make an immediate diagnosis of Sprengel's deformity of the shoulder, a dysplasia of the pectoral girdle that causes cosmetic and functional disability. This case report serves as a reminder of the importance of continued research, education, and collaboration in the field of paediatrics and orthopaedics, enhancing us to better address congenital anomalies like Sprengel's deformity.
